# Integrative analysis reveals disease-associated genes and biomarkers for prostate cancer progression

**DOI:** 10.1186/1755-8794-7-S1-S3

**Published:** 2014-05-08

**Authors:** Yin Li, Wanwipa Vongsangnak, Luonan Chen, Bairong Shen

**Affiliations:** 1 Center for Systems Biology, Soochow University, Suzhou, 215006, China; 2 Key Laboratory of Systems Biology, Chinese Academy of Sciences, Shanghai, 200031, China

**Keywords:** Biomarker, Disease-associated Genes, Integrative analysis, Prostate cancer, Transcription factor

## Abstract

**Background:**

Prostate cancer is one of the most common complex diseases with high leading cause of death in men. Identifications of prostate cancer associated genes and biomarkers are thus essential as they can gain insights into the mechanisms underlying disease progression and advancing for early diagnosis and developing effective therapies.

**Methods:**

In this study, we presented an integrative analysis of gene expression profiling and protein interaction network at a systematic level to reveal candidate disease-associated genes and biomarkers for prostate cancer progression. At first, we reconstructed the human prostate cancer protein-protein interaction network (HPC-PPIN) and the network was then integrated with the prostate cancer gene expression data to identify modules related to different phases in prostate cancer. At last, the candidate module biomarkers were validated by its predictive ability of prostate cancer progression.

**Results:**

Different phases-specific modules were identified for prostate cancer. Among these modules, transcription Androgen Receptor (AR) nuclear signaling and Epidermal Growth Factor Receptor (EGFR) signalling pathway were shown to be the pathway targets for prostate cancer progression. The identified candidate disease-associated genes showed better predictive ability of prostate cancer progression than those of published biomarkers. In context of functional enrichment analysis, interestingly candidate disease-associated genes were enriched in the nucleus and different functions were encoded for potential transcription factors, for examples key players as AR, Myc, ESR1 and hidden player as Sp1 which was considered as a potential novel biomarker for prostate cancer.

**Conclusions:**

The successful results on prostate cancer samples demonstrated that the integrative analysis is powerful and useful approach to detect candidate disease-associate genes and modules which can be used as the potential biomarkers for prostate cancer progression. The data, tools and supplementary files for this integrative analysis are deposited at http://www.ibio-cn.org/HPC-PPIN/.

## Background

Prostate cancer is the second leading cause of morbidity and mortality in men [1,2]. In recent years, the incidence rate of prostate cancer has dramatically increased [3], and this is largely because of lack of diagnosis and treatment of the disease at the early stage [4]. Thus, the successful clinical biomarkers for early diagnosis of the presence of prostate cancer become very urgent to reduce the death risk of the prostate cancer [5,6].

In the post-genomics era, there is an explosion of biological data and information generated from high-throughput technologies which have rapidly provided an unprecedented multi-level omics data [7]. Such transcriptomics, referred to as gene expression profiling can now comprehensively survey the entire human genomics. Moreover, enormous efforts have been made to identify biomarkers for various cancers by the analysis of different transcriptomics data [8-12]. As an example reported by our previous study, integrative transcriptomics data could be used to identify putative novel prostate cancer associated pathways, such as Endothelin-1/EDNRA trans-activation of EGFR pathway which would provide essential information for development of network biomarkers and individualized therapy strategy for prostate cancer [11-13]. Looking at the other relevant studies for cancer transcriptomics, a large scale expression study presented by Wang et al. identified a set of gene markers for prediction of metastasis for breast cancer [14] and followed by Chari et al. demonstrated an approach based on multiple concerted disruptions (MCD) analysis and identified genes and pathways in cancer [15]. Furthermore, transcriptomics could be used to identify metabolic biomarkers through alterative metabolic pathways at different cancer phases [16]. Concerning on the other levels of omics, proteomics in context of protein-protein interaction network could also be used to characterize and diagnose a pathological process [17]. As clearly reported by Ideker and Sharan [18], the indicating genes as biomarkers in complex diseases tend to cluster together on well-connected proteins interaction sub-networks. In following years, Chuang et al. also showed that it could be useful to extract co-expressed functional sub-networks for metastasis of breast cancer through integrating transcriptomics data with protein-protein interaction to obtain higher classification accuracy [19]. Later, Taylor et al. studied the altered protein interaction modularity to predict breast cancer progression by examining the biochemical structure of the interactome [20]. Besides, there were similar studies for analysis of sub-networks and/or hub proteins which had been helpful for the understanding of the metastasis of cancer at the molecular level [18].

Focusing on prostate cancer, there were some reports on identifying disease-related gene modules, sub-networks or dysfunctional pathways focused on global characteristics of interactome together with gene expression data by different novel algorithms and methods development [21-23]. Nonetheless, there are still few studies on identification of prostate cancer biomarkers for early detection of the presence as well as disease progression [20]. The relationships among the potential prostate cancer genes and associated functions as well as pathways are still poorly characterized, such as how they interacted and regulated with each other, also what they act within the network modules. These investigations are warranted for a comprehensive understanding of the molecular mechanisms underlying prostate cancer progression. Hence, it is a challenge to perform an integrative analysis of different data, which can be gene expression profiling, protein-protein interaction (PPI) data, pathway information, and clinical information, that can offer different perspectives on the biological problems in prostate cancer and further identification of potential biomarkers [24,25].

In this study, we therefore aim to reveal candidate disease- associated genes and biomarkers for prostate cancer progression by integrative gene expression profiling and network analysis at a systematic level. We first reconstructed human prostate cancer protein-protein interaction network and used this network as a scaffold for further integrative analysed with gene expression data of prostate cancer. Here, analysis of gene expression profiling of prostate cancer was performed at different disease phases. Through modular analysis, the different modules associated with disease phases were then identified. Last but not least, we could identify significant genes through these modules which were supposed to be the gene expression signatures with highly relevant to specific phases of prostate cancer. Once the common genes identified in each of different modules were overlapped, expectedly these common genes were beneficial for uncovering of novel prostate cancer-related pathways and transcription factors which could be candidate biomarkers for prostate cancer progression. Our study hereby demonstrated a practical workflow for integrative analysis of prostate cancer at the systematic level. For the genome-wide studies, this will be a basic effort for future development and evolution in aspects of the translational biomedical informatics, which ultimately intend to improve patient outcomes and diagnostics with omics dataset through integrative systems biology [26].

## Methods

### Human prostate cancer protein interaction network reconstruction and annotation

**Figure 1:**
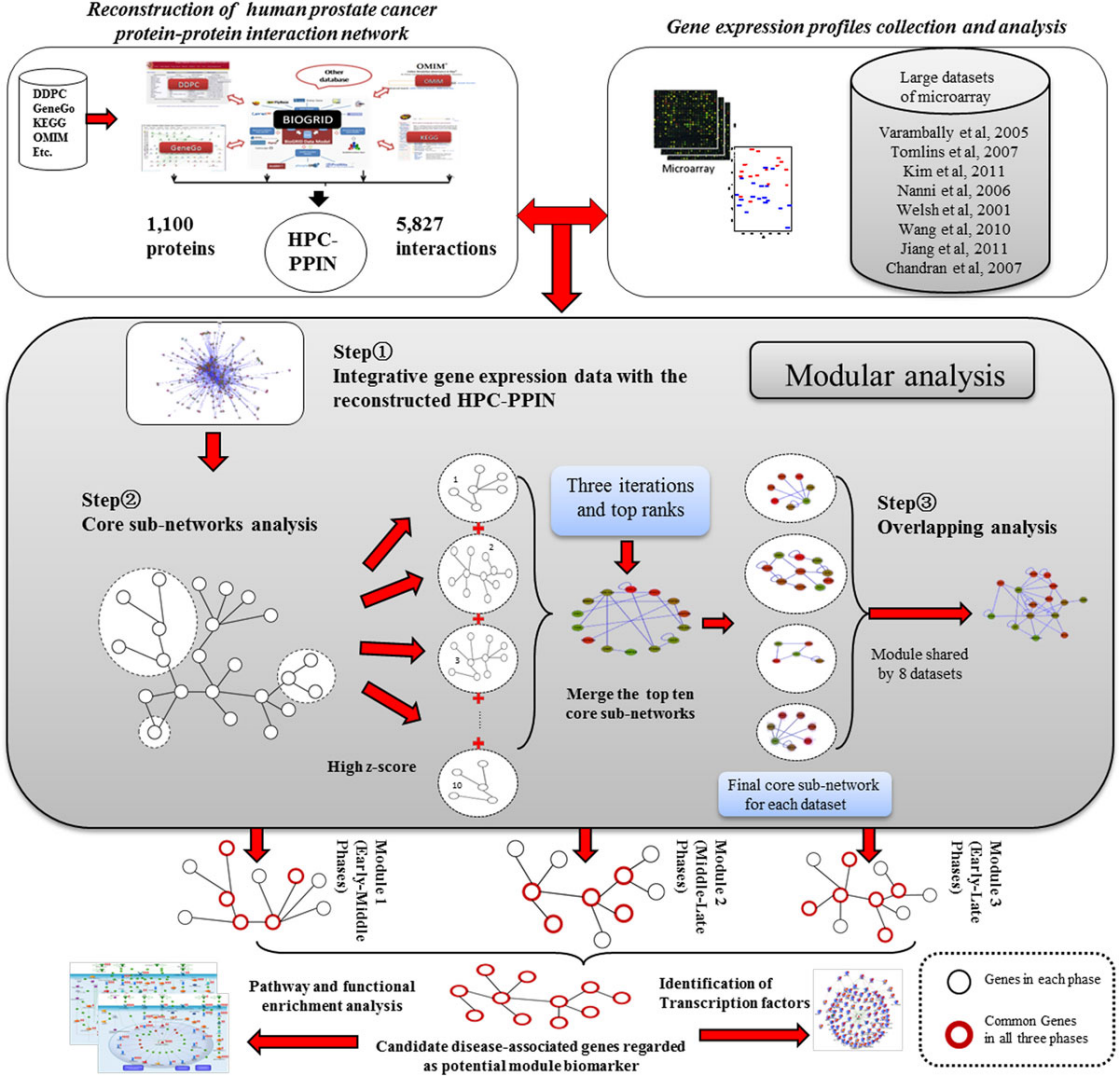
**The modular analysis pipeline**. Diagram shows identification of candidate disease-associated genes as potential module biomarker based on integrative analysis of the reconstructed human prostate cancer protein-protein interaction network (HPC-PPIN) and the different phases of gene expression profiles of prostate cancer. The threshold for greedy algorithm via Cytoscape jActiveModules (jAM) plugin for the most significant core sub-networks analysis in each gene expression profile was set to three iterations and top ten ranks.

The human prostate cancer protein-protein interaction network (HPC-PPIN) was initially reconstructed in order to be further used for integrative analysis as a diagram illustrated in Figure 1. To reconstruct the HPC-PPIN, we used two different types of datasets. The first dataset was the genes associated in prostate cancer derived from a collection of prostate cancer databases and other relevant resources (e.g. Dragon Database of Genes associated with Prostate Cancer (DDPC) [27], GeneGo [28], OMIM [29], KEGG [30], PGDB [31], CCDB [32], and Gene Ontology (GO) [33]).

For the second type of the dataset, it was the human protein-protein interactions data (Homo sapiens) which was downloaded from the BioGRID database [34]. Concerning on annotation of the HPC-PPIN, we used the Database for Annotation, Visualization and Integrated Discovery (DAVID) system [35,36]. At the beginning, functional annotation clustering tool of DAVID system was applied to group annotated genes within HPC-PPIN across three GO processes underlying molecular function, biological process, and cellular component. Among three GO processes, this tool was then used to identify the enriched GO terms. In order to annotate detailed functions in context of pathways underlying metabolism, cellular process, environmental information process and genetics information process, KEGG database was used (http://www.genome.jp/kegg/pathway.html).

### Prostate cancer gene expression data collection and analysis

The gene expression profiles based different platform arrays from different stages of prostate cancer (i.e. disease stages I, II, II, IV) were collected from various laboratories. Table 1 lists available information of collected gene expression profiles (431 samples) of prostate cancer progression. Since only fewer samples are available in stage I than other disease stages, stages I and II were combined into one phase (Table 1). All expression datasets were analysed for gaining statistics values. The statistical processing methods were invoked through the limma (Linear Models for Microarray Data) package in R [37,38] and scripting under R version 2.9.0 (R Development Core Team). The limma package [37] was applied to perform moderated Student's t-test between all possible pairwise disease phases comparison i.e., early-middle phases, middle-late phases, and early-late phases, to determine significantly differential gene expression. Empirical Bayesian statistical method was applied to moderate the standard errors within each gene and then the Benjamini-Hochberg's method was applied to adjust the multi-testing [39], as well as to obtain the adjusted p-value.

**Table 1:** Gene expression profiles of prostate cancer used for integrative analysis#

**No. Exp**.	Platform		No. Probes	Samples Series	No. Samples of prostate cancer stages				References
					***NAP***	***BN***	***PCA***	***MET***	
					***(I)***	***(II)***	***(III)***	***(IV)***	
						
					***Early phase***		***Middle phase***	***Late phase***	

Exp-1	Affymetrix HG-U133P2	GPL570	54,675	GSE3325	0	6	7	6	[76]
Exp-2	cDNAChinnaiyan Human 20K Hs6	GPL2013	20,000	GES6099	0	15	32	20	[77]
Exp-3	Agilent-014850 4x44K G4112F	GPL4133	45,220	GSE27616	0	2	5	4	[78]
Exp-4	Affymetrix HG-U133A	GPL96	22,283	GSE3868	2	0	22	0	[79]
Exp-5	Affymetrix HGU95A	-	12,626	-	9	0	25	0	[80]
Exp-6	Affymetrix HG-U133P2	GPL570	54,675	GSE17951	0	45	109	0	[81,82]
Exp-7	Agilent-014850 4x44K G4112F	GPL6480		GSE28204	0	4	4	0	[83]
Exp-8	Affymetrix HG_U95Av2	GPL8300	12,625	GSE6919	18	0	65	25	[84,85]

#Abbreviations: NAP: normal prostate samples, BN: benign samples, PCA: primary prostate cancer samples, MET: metastatic prostate cancer samples

### Modular analysis for prostate cancer progression

In order to perform modular analysis for study of disease progression, three main steps were necessarily performed. At the first step, the analysed gene expression data previously derived from pairwise disease phase comparison of prostate cancer was integrated with the reconstructed HPC-PPIN. Hereafter core sub-networks analysis and overlapping analysis as second and the third steps were then performed, respectively. Regarding on the core sub-networks analysis, they were investigated for which were shown highly active scores and top ranks based on the greedy algorithm. In this investigation, the greedy algorithm was selected for searching the core sub-networks in a large network of interactions from any pairwise disease phases comparison, where refers to a connected sub-graph of the interactome that has high significance of differential expression values [19]. To elaborate how the greedy algorithm used, originally the adjusted p-value derived from any pairwise disease phases comparison was converted to the readily form of z-score by using the inverse normal cumulative distribution (θ^-1^) for scoring and ranking [40]. Afterwards the greedy algorithm by jActiveModules (jAM) plug-in as implemented in the Cytoscape [41,42] was used to investigate and extract the significant core sub-networks under threshold of three iterations and top ten ranks. Through the end, the list of top ten ranks were merged together to gain a final core sub-network which represented for each of pairwise disease phases comparison and for each of gene expression profile. Notably, jAM was chosen as a basis for this investigation because it is a fashionable method, based on a survey of the current literature. There are several successful cases where jAM has been applied to extract the significant core sub-networks, for examples in fruit fly Drosophila [43], yeast S. cerevisiae [44], worm C. elegans [45] and human H. sapiens [19,46].

To finalize the modular analysis, the overlapping analysis was carried out. The overlapping analysis at gene level was applied to show the number of enriched genes shared by all gene expression profiles (see Table 1) calculated based on core sub-networks analysis. For example, considering each of a final core sub-network retrieved from each of pairwise disease phase comparison analysing across all gene expression profiles, the overlapping percentage of genes was calculated between any two of the final core sub-networks derived from any two of the gene expression profiles. For the formula of the overlapping analysis, we defined the number of genes

in the final core sub-network1 as (CS^1^) and the final core sub-network2 as (CS^2^). The overlapping percentage between the final core sub-networks was designated as the number of overlapping genes (G) divided by the number of genes in the union of (CS^1^) and (CS^2^) with subtracted (G). It can be calculated as follows in following formula (1):

(1) $$Overlapping\;percentage=\frac{G}{CS^{1}+CS^{2}-G}\times 100\%$$

After overlapping analysis, as a result the overlapping percentage across all gene expression profiles was obtained for each of pairwise disease phase comparison. Towards all possible pairwise disease phases comparison (i.e. early-middle phases, middle-late phases, and early-late phases), three different modules associated with disease progression were eventually identified. It is very possible that each of these three modules plays important roles in dynamic changes of molecular interactions at a specific phase of the disease progression. The identified unique genes in each module were regarded as signatures at a specific phase of prostate cancer. The identified common genes in all three modules were regarded as candidate disease-associated genes.

### Identified candidate prostate cancer associated genes as putative module biomarker

To validate the identified prostate cancer associated genes as putative module biomarker, we used them as a module biomarker to discriminate between control and prostate cancer samples. Support vector machine (SVM) regression proposed by Cortes and Vapnik [47] was selected due to its attractive features and high performances [48-50] for applying to the expression values of the predicted prostate cancer associated genes from the module biomarker to distinguish prostate cancer from controls. The Receiver Operating Characteristic (ROC) curve and the area under curve (AUC) were used to evaluate the efficiency of classification [51-53]. Two R packages, namely kernlab [54] and ROCR [55], were applied to build the SVM classifier and produced the ROC curves.

### Validation of candidate prostate cancer associated genes by statistical methods

For the validation of candidate prostate cancer associated genes, known related genes obtained from the Cancer Gene Census database [56] (accessed on December 6, 2012), Genetic Association Database (GAD) [57] (accessed on October 27, 2012) and AnimalTFDB [58] (accessed on December 7, 2012) were used. A hypergeometric test was applied to estimate the enrichment of these candidate prostate cancer associated genes compared to the known cancer related genes. The equation of the hypergeometric test is shown as follows in (2):

(2) $$P\left(X\geq x\right)=1-\overset{x-1}{\underset{k=0}{\sum }}\frac{\left(\begin{array}{c} M\\ k \end{array}\right)\left(\begin{array}{c} N-M\\ n-k \end{array}\right)}{\left(\begin{array}{c} N\\ n \end{array}\right)}$$

In the above equation, N and M represents the number of genes in the expression profiles and the number of known cancer genes respectively, n and k are the number of the candidate prostate cancer associated genes that we identified, and the number of common entries between them, respectively. P represents the statistical significance of the enrichment. Random sampling was used to test the statistical significance and the same number of known cancer genes was randomly selected from Cancer Gene Census database [56], Genetic Association Database (GAD) [57] and AnimalTFDB [58] to assess the statistically significance of these known cancer genes included in the previous results. At first, the same number of genes as the candidate prostate cancer associated genes was randomly selected from the reconstructed HPC-PPIN. Subsequently, the number of known cancer genes included in the random samples was then counted. Afterwards, random sampling was repeated 10^6 ^times. Then, the p-value of the candidate prostate cancer associated genes was defined as the probability that one random sampling might contain a greater or equal number of known cancer genes than in our study samples.

### Functional and pathway enrichment analysis

The GeneGo, which is a commercial integrated knowledge database [59], was used for analysis of functional and pathway enrichment. The statistical significance value was calculated using hypergeometric distribution and false discovery rate (FDR) method (p value < 0.05).

## Results and discussion

### Reconstructed HPC-PPIN and its functional annotation

The HPC-PPIN was reconstructed from different prostate cancer databases and other relevant resources along with one directional interaction and repeat interactions removed, hereafter resulting in 5,827 interactions among 1,100 proteins. The characteristics of the reconstructed HPC-PPIN are shown in Table 2. Additional file 1**(Figure S1) **illustrates the distributing numbers of prostate cancer- related genes from different databases with assigned and unassigned in HPC-PPIN.

**Table 2:** Number of genes, proteins and interactions between pairwise disease phases comparison#

Features	Early-Middle phases	Middle-Late phases	Early-Late phases
Number of proteins in the reconstructed HPC-PPIN	1,100	1,100	1,100
Number of genes within analyzed modules	217	193	266
Number of interactions within analyzed modules	1,832	1,564	1,787
bNumer of candidate disease-associated genes	94	94	94

#Abbreviations: HPC-PPIN: Human prostate cancer protein-protein interaction network

**Figure 2:**
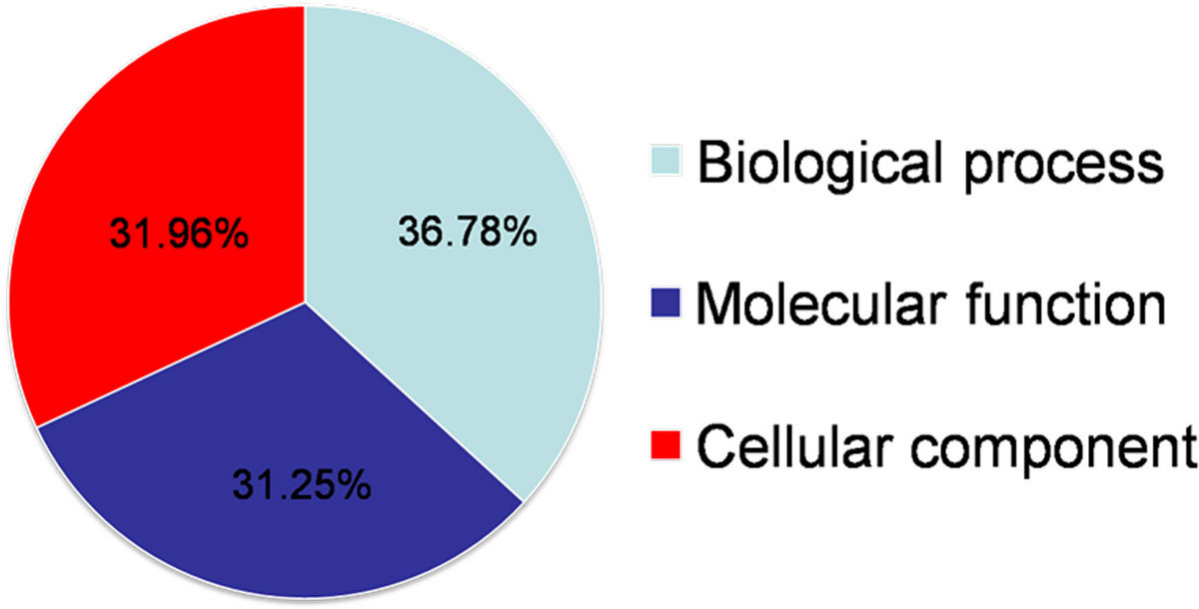
**Annotated functions for the reconstructed HPC-PPIN using DAVID system**. Pie-chart shows different frequencies of three GO processes distributing into HPC- PPIN.

**Figure 3:**
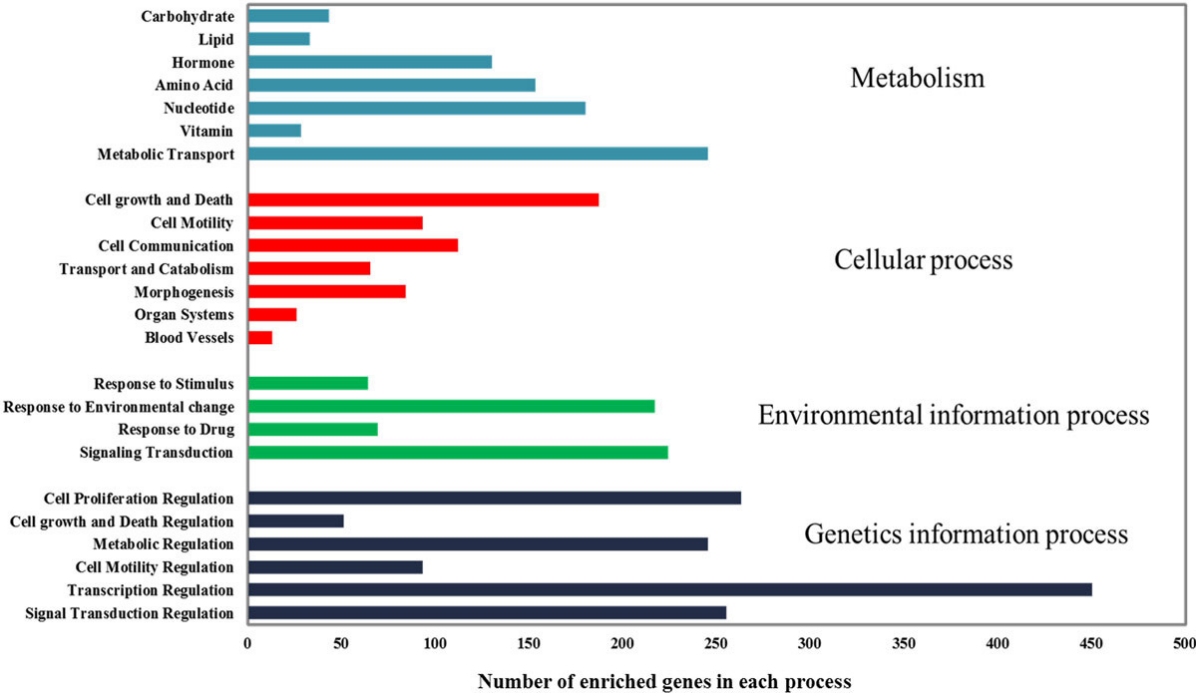
**Annotated functions for the reconstructed HPC-PPIN using DAVID system**. Bar graph presents different functional classifications distributing into HPC-PPIN based on KEGG categories.

Concerning on the annotated functions of HPC-PPIN in DAVID system, we found that the major of GO terms involved in biological process (36.78%) as illustrated in Figure 2. Considering to KEGG categories, we found that the major annotated functions were involved in genetics information process in the category of transcription regulation (41%). The results are shown in Figure 3. The reconstructed HPC-PPIN with annotated functions is shown in Additional file 2. A graphic representation of HPC-PPIN by Cytoscape [42] is presented in Additional file 3.

### Modules involved in prostate cancer progression

**Figure 4:**
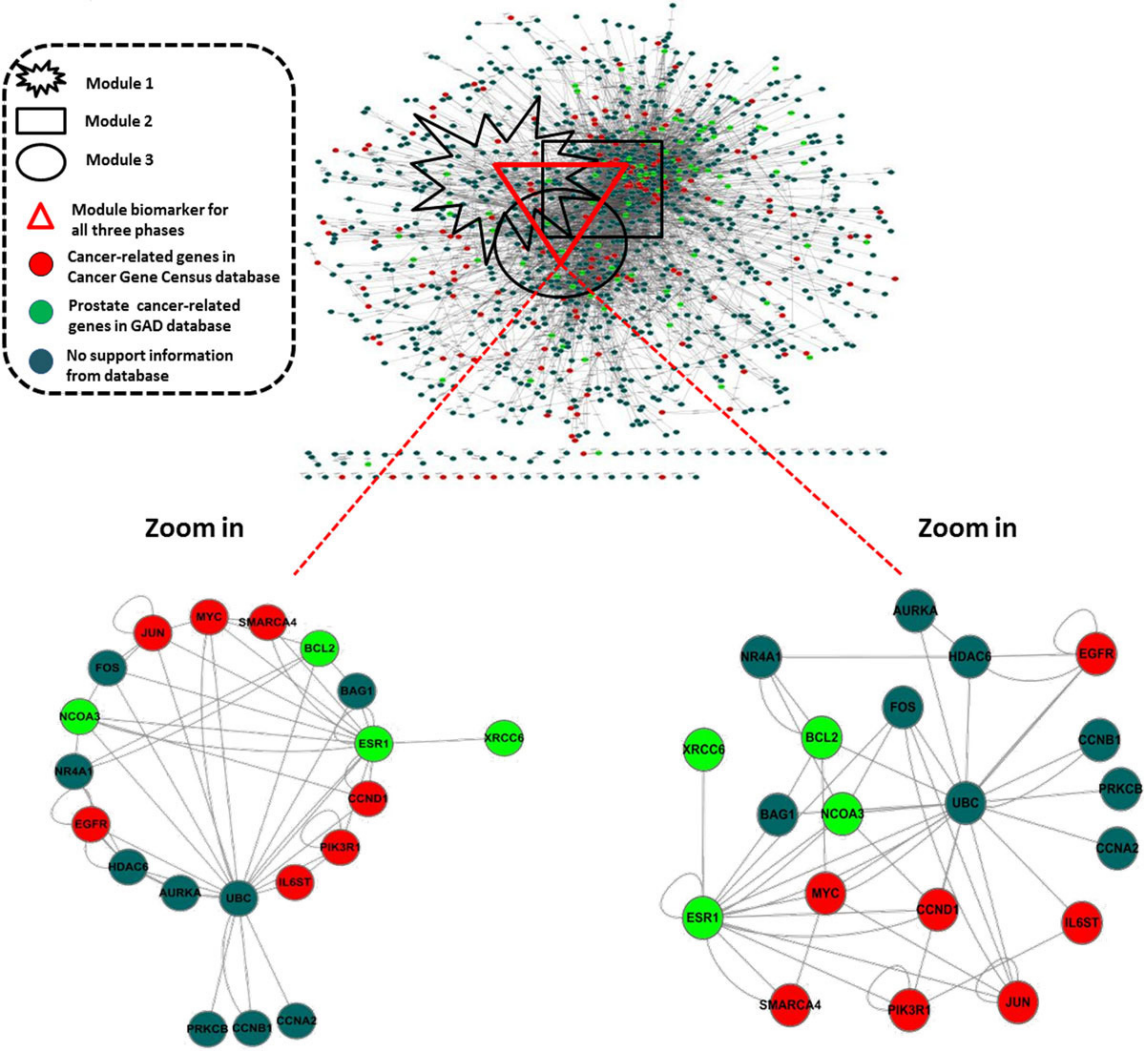
**The interaction network of three modules obtained from modular analysis for prostate cancer progression**. The highlight interactions within the 94 candidate disease-associated genes identified in all three phases were obtained. The red node indicates cancer-related genes in Cancer Gene Census database. The green node indicates prostate cancer- related genes in GAD database. The blue node indicates no support information from database.

As described in modular analysis for prostate cancer progression (Section in Materials and Methods), three different modules were obtained. The results are shown in Figure 4. As presented in the Table 2, noticeably the third module underlying early-late phases comparison contained the maximum number of genes (266 genes), in contrast to the second module underlying middle-late phases comparison contained the minimum number of genes (193 genes). This suggests that the third module has additional gene expression signature changes (82 genes) than the other two modules identified for 56 genes in the first module and 30 genes in the second module. These can be explained that cancer cells possibly develop new mechanisms and regulations for cell proliferation from an initial stage and further enhance tumour metastasis with degenerative disease. Additional file 1 (Table S1) lists all unique gene expression signatures identified in each module.

To further elaborate functions of unique gene expression signatures, literature search using PubMed was performed. Our finding clearly showed that unique gene expression signatures play important roles in progression of prostate cancer at a specific phase. For examples, SMAD3 and TGFB2 were reported as androgen- independent prostate cancer-specific genes [60] which were found in a specific expression of early-late phase. In addition, there were more unique gene expression signatures in early-late phase, for instances PTEN, BRAF, DDX5, NCOA4, WHSC1, CCND2, CDH11, ERCC5, FANCD2, LIFR, MAF, RAF1, and TOP1. Examples of unique gene expression signatures in middle-late phase, we found TP53 and RB1 which were reported as tumour suppressor genes. Growing evidences were also shown in transcription factor, such as STAT3 which was identified only in early-middle phase.

**Figure 5:**
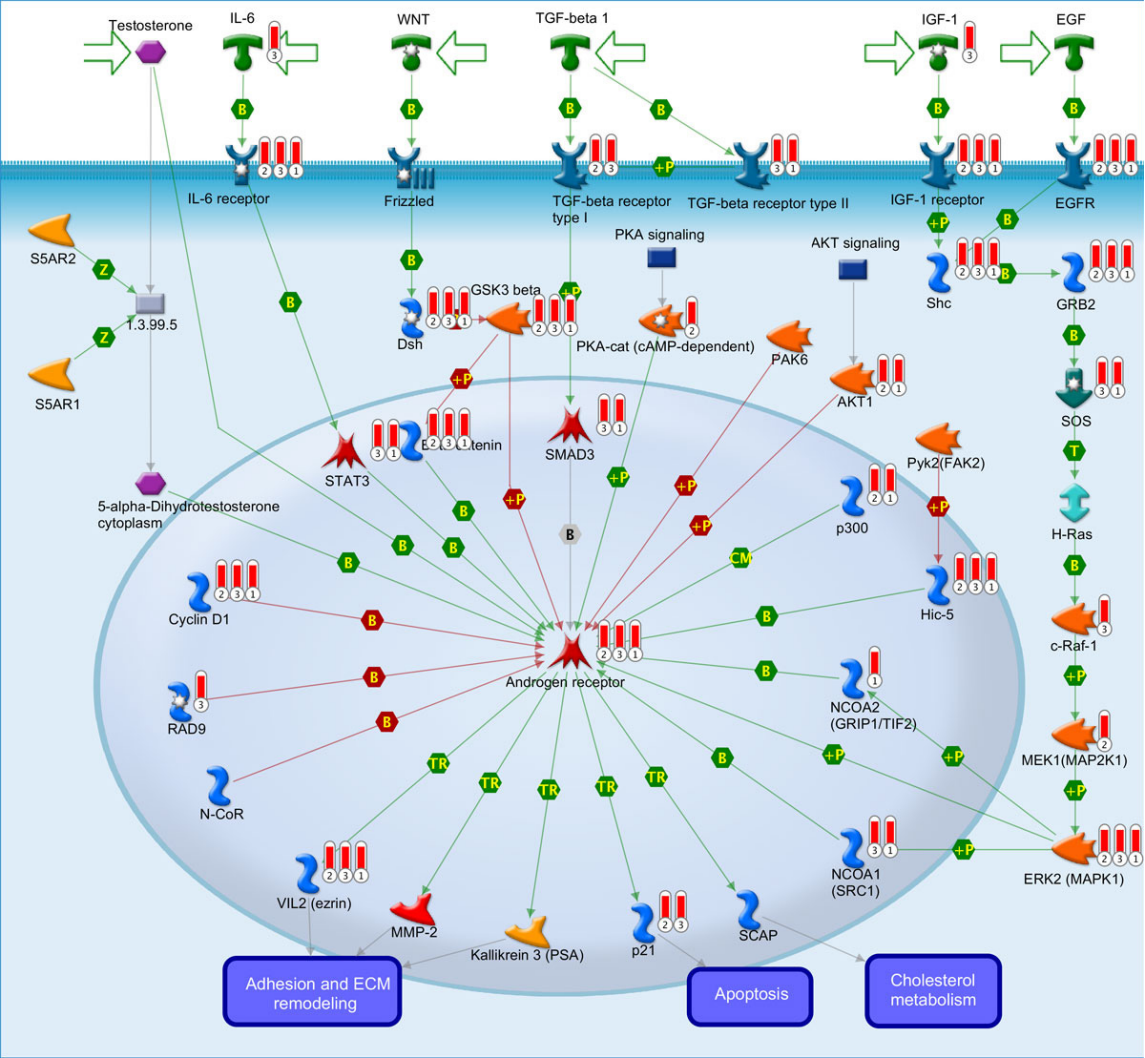
**GeneGo graphic representation illustrates Transcription Androgen Receptor (AR) nuclear signaling**. Pathway regarded as the enriched significant pathway associated in prostate cancer progression. Transcription factor AR was identified as a hub protein to play a critical role in prostate tumorigenesis and prostate cancer progression.

Regarding on pathway enrichment analysis associated in prostate cancer, interestingly Transcription Androgen Receptor nuclear signaling was found to be the enriched pathway as illustrated in Figure 5. Obviously, Transcription factor AR plays an important role in Transcription relationship which was found in all three modules and appeared to be a hub for regulating a lot of genes in this pathway. We also showed the other enriched pathways as shown in Additional file 1 (Fig. S2), for example Development Epidermal Growth Factor Receptor (EGFR) signaling pathway as presented in Additional file 1 (Fig. S3). As known, EGFR signalling pathway regulates cell proliferation, cell differentiation, cell cycle, and cell migration. Undoubtedly, EGFR pathway therefore becomes a part of a complex network that has been an interested target for effective cancer therapies [61,62]. From this study, the results also showed consistency with our previous work [12].

### Candidate disease-associated genes in prostate cancer progression and their statistical significance

**Figure 6:**
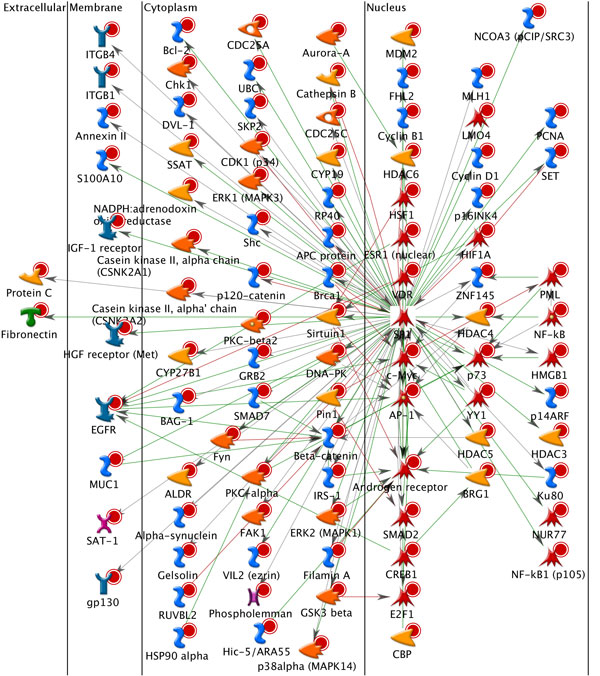
**GeneGo graphic representation for module biomarker**. It shows a major fraction of 94 candidate disease-associated genes which was enriched in the nucleus and different functions were mostly encoded for transcription factors.

In order to identify candidate disease-associated genes in prostate cancer progression, the gene members in each module were overlapped among three modules. With repeat interactions removed, 94 genes were found as the common members and regarded as the candidate disease-associated genes (see Figure 3 and Table 2). For biological interpretation, functional enrichment analysis of our candidate disease-associated genes was conducted using GeneGo [28]. Based on different sub-cellular localizations, namely extracellular, membrane, cytoplasm, and nucleus, a major fraction of 94 candidate disease-associated genes was enriched in the nucleus and different functions were mostly encoded for transcription factors as illustrated in Figure 6.

Additionally, we compared the candidate disease-associated genes with public databases as shown in Table 3. As a result, 23 out of 94 genes were found in Cancer Gene Census database, and 22 out of 94 genes were identified as prostate cancer- related genes from GAD, as well as 18 out of 94 genes were recognized as transcription factors from AnimalTFDB. Concerning on high degree of interactions (≥10 interacted genes), 15 out of 23 genes (65.2%) were found to be hubs when Cancer Gene Census database was used. For other databases, GAD and AnimalTFDB showed 18 out of 22 genes (81.8%) and 12 out of 18 genes (66.7%) were found to be hubs, respectively. These results suggest that cancer-related genes and transcription factors likely showed to be the hub genes. In addition, self-interacting genes tended to be cancer-related genes and transcription factors (shown in Figure 4). As evidences subsequently presented, 32 out of 94 genes as self-interacting genes were found to be 10 cancer-related genes based Cancer Gene Census database, 9 prostate cancer-related genes based GAD, and 9 transcription factors based AnimalTFDB.

**Table 3:** Summary of statistical significance of candidate disease-associated genes in prostate cancer progression#

Database	Known reported genes	Genes in the HPC-PPIN	Candidate disease-associated genes	HD, RS (P-values)
Cancer Gene	488	129	23	6 × 10^-4^,
Census				<3 × 10^-4^
GAD	309 (prostate cancer only	155	22	7 × 10^-3^, 7 × 10^-3^
AnimalTFDB (Transcription factors)	1,457	121	18	9 × 10^-3^, 7 × 10^-3^

#Abbreviations: HD: Hypergeometric Distribution, RS: Random Sampling

To further assess statistical significance of 94 candidate disease-associated genes, the Cancer Gene Census database [56], GAD [57], and AnimalTFDB [58] were also used. 23 out of 94 genes underlying 488 genes which have been reported to be related to cancer in Cancer Gene Census database [56], we further investigated whether these genes could be randomly obtained. Statistical significance was checked using hypergeometric distribution and 106 times random simulation. The results showed that two significant p-values of 6 × 10^-4 ^and < 3 × 10^-4 ^were obtained, respectively. These indicate that the candidate cancer genes are enriched among known cancer-related genes and cannot be obtained randomly. For GAD [57], 22 out of 94 genes underlying 309 genes reported to be related to prostate cancer. The statistical significance was similarly checked. Two significant p-values of 7 × 10^-3 ^from a hypergeometric distribution and7 × 10^-3 ^from random simulation were obtained. Once using the AnimalTFDB [58], 18 out of 94 genes underlying 1,457 genes, which have been reported to be related to transcription factors. Statistical significance was similarly checked. As a result, two significant p-values of 9 × 10^-3 ^and 7 × 10^-3 ^were obtained with a hypergeometric distribution and random simulation, respectively.

### Validation of candidate disease-associated genes regarded as potential module biomarker

**Figure 7:**
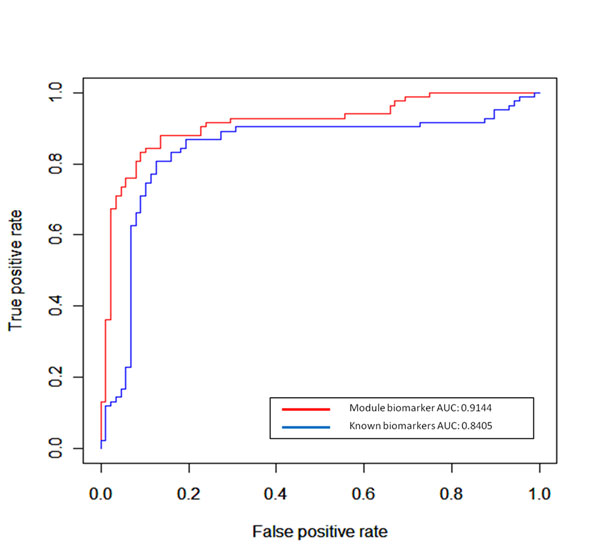
**ROC curves are obtained with our module biomarker and known biomarkers**. The gene expression dataset (series of GSE6919) from GEO database (www.ncbi.nlm.nih.gov/geo//)

**Figure 8:**
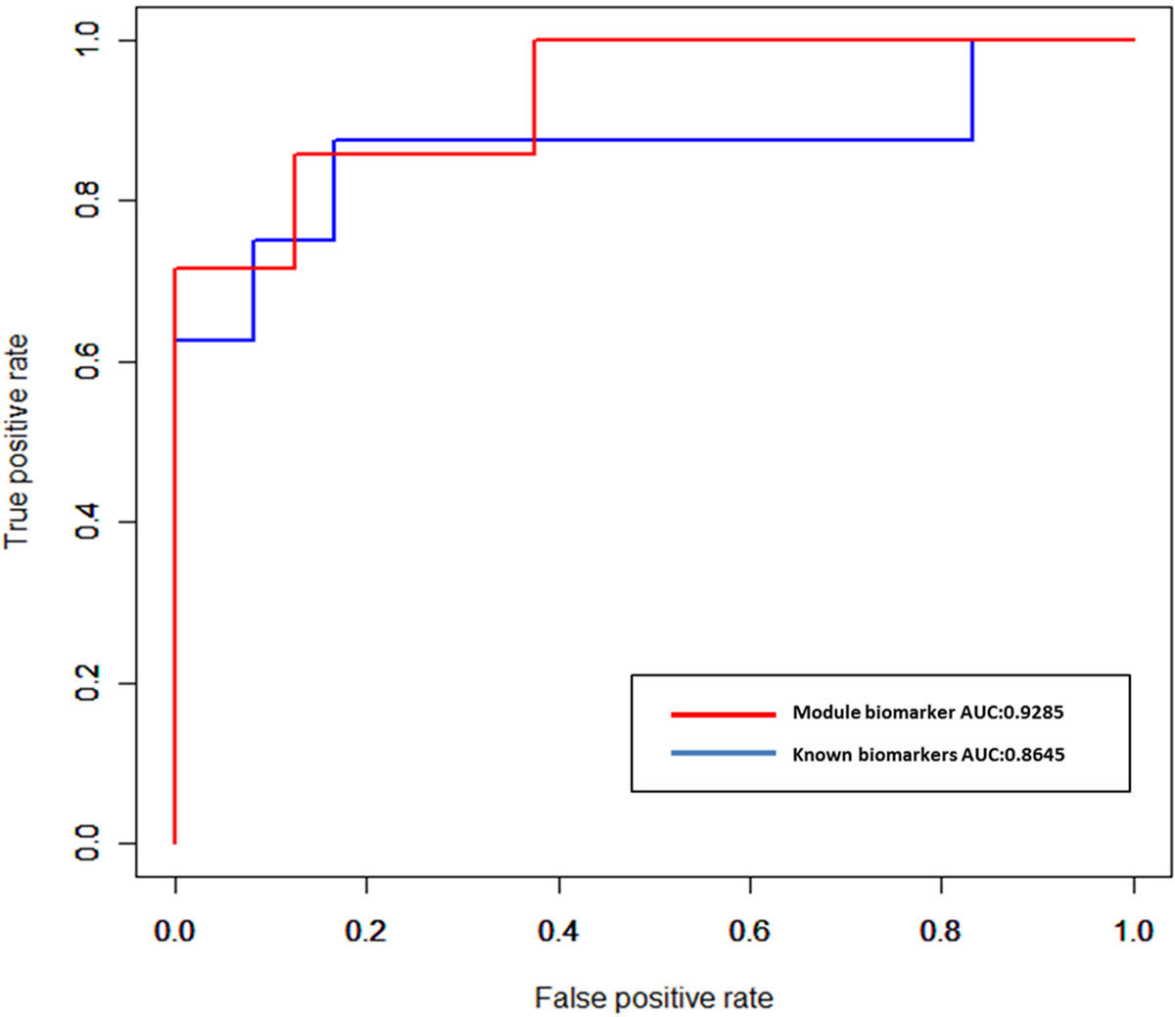
**ROC curves are obtained with our module biomarker and known biomarkers**. The independent gene expression dataset[75]. AUC means area under curve and ROC means receiver operating characteristic.

To further validate the ability of the candidate disease- associated genes to distinguish cancer samples from controls, the gene expression dataset in series of GSE6919 for prostate cancer obtained from GEO database (http://www.ncbi.nlm.nih.gov/geo/) and the independent gene expression dataset [63] were used as the tested datasets. Here, we hypothesized that if our candidate disease-associated genes can successfully distinguish cancer samples from control samples in these tested datasets, they can be further shown to be related to prostate cancer and regarded as a potential module biomarker. Moreover, we compared our results with those obtained with a public biomarker set for prostate cancer [64], which were derived from differential gene expression. Five-fold cross validation was used to assess the performance based on different biomarkers and the SVM regression was used as the classifier. Figure 7 and 8 show the ROC curves obtained with our candidate disease-associated genes as module biomarker and known biomarkers individually for these two tested datasets. In addition, we also show AUC (area under curve) to provide the statistical summary of the performance of the classification over the entire range of sensitivity and specificity. In Figure 7, for the GSE6919 gene expression dataset, our module biomarker shows AUC of 91.44% and known biomarkers show AUC of 84.05%. In Figure 8 for the independent gene expression dataset [63], our module biomarker and known biomarkers show AUC of 92.85% and 86.45%, respectively. These results confirm that our identified putative prostate cancer associated module biomarker performs well with respect to the known biomarkers; therefore it could be potentially applied to predict prostate cancer progression.

### Transcription factor Sp1 as a novel biomarker for prostate cancer

Towards candidate disease-associated genes as the potential module biomarker, interestingly, we found 18 key transcription factors which had a major fraction involved in Transcriptional regulation. Accordingly, it is possible that these key transcription factors probably regulate a large number of genes and are called potential candidates to be biomarkers for prostate cancer. This is based on the concept that transcription factors are the drivers of the potential regulation of genes in prostate cancer, and thus are relevant for use as biomarkers [65].

In order to identify potential candidates to be biomarkers for prostate cancer, we initially mapped 94 candidate disease-associated genes to GeneGo which invoked an appropriate algorithm to build networks relevant to active data, such as our gene list in a straightforward manner depending on the task. Later, we chose Transcription regulation workflow from GeneGo which generated sub-networks centred on transcription factors. Sub-networks were then ranked by p-values and interpreted in terms of gene ontology. Afterwards, a few of sub-networks containing receptors with direct ligands from our datasets and their closet transcription factors that directly targeted the objects with these datasets were generated. To the end, we could identify potential transcription factors regarded as candidates to be biomarkers for prostate cancer which had a Transcription regulation relationship with the regulated candidate disease-associated genes.

**Figure 9:**
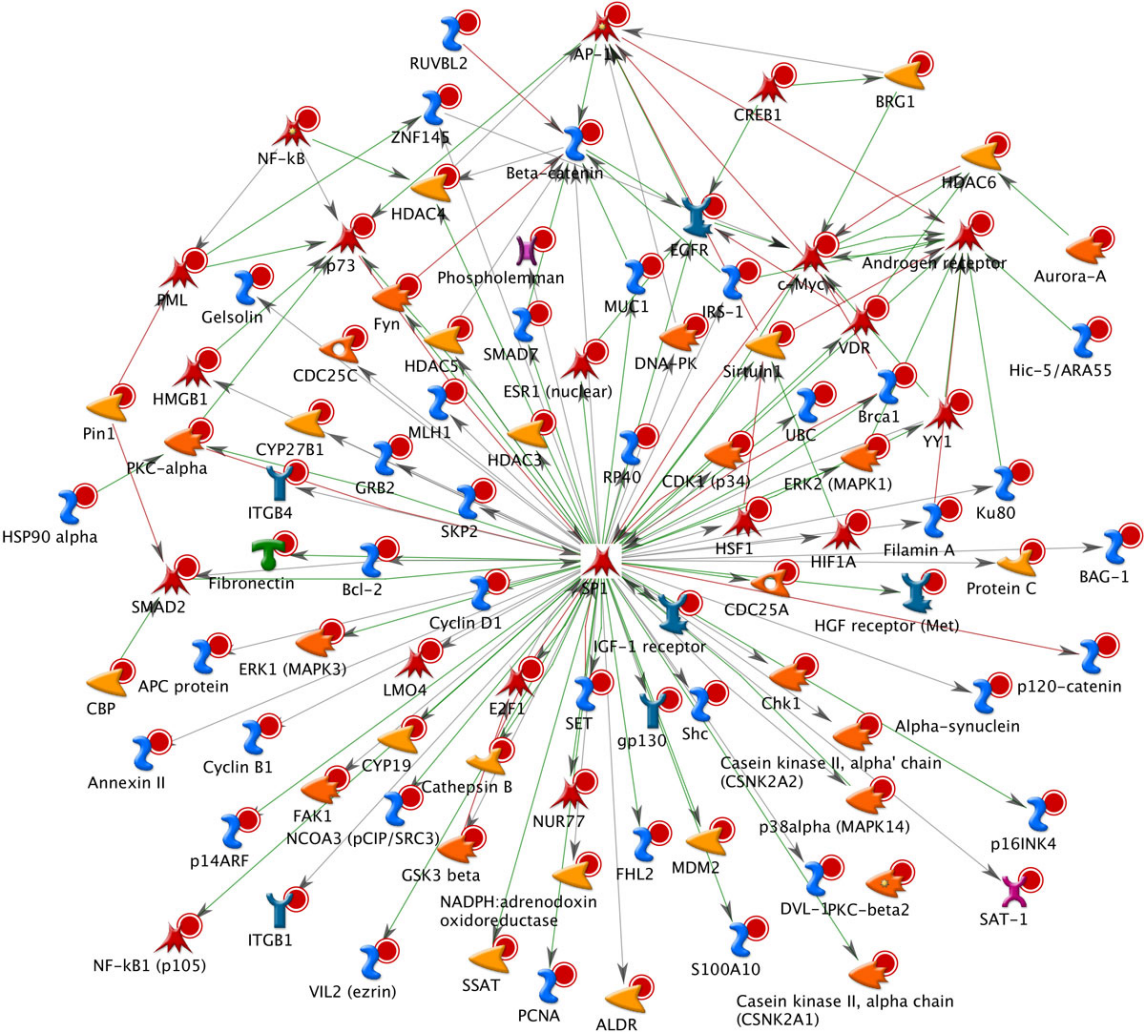
**GeneGo graphic representation illustrates transcription factor Sp1 as a potential novel biomarker of prostate cancer**. Sp1 is a hidden key transcription factor which directly regulates a lot of candidate disease-associated genes, and also has indirect effect with the remaining genes.

Successfully, we found Myc, AR, ESR1 and p53 as potential transcription factors which were possibly regarded as biomarkers for prostate cancer as shown in Additional file 1 (Figure S4) [66-69]. Surprisingly, our identification showed that Specificity Protein 1 (Sp1) was a hidden key transcription factor involved in regulation of gene expression in early development of human prostate cancer [70]. We found that transcription factor Sp1 directly regulated a lot of candidate disease- associated genes, and also had indirect effect with the remaining genes. The result shows in Figure 9. Focusing on prostate cancer studies, several reports have shown that transcription factor Sp1 regulates some important genes like androgen receptor (AR) and TGF-β [71-73]. Moreover, transcription factor Sp1 has also been found as a new biomarker that could identify a subset of pancreatic ductal adenocarcinoma with aggressive clinical behaviour. It can be used at initial diagnosis of pancreatic adenocarcinoma to identify patients with an increased probability of cancer metastasis and much shortened overall survival [74]. As many articles reported that transcription factor Sp1 plays an important role with clinical behaviour and it is identified as a hub around different transcriptional changes. We therefore propose that transcription factor Sp1 is probably a novel candidate diagnosis biomarker related to prostate cancer. We expect that the future application of transcription factor Sp1 as a biomarker for prostate cancer may improve clinical management.

## Conclusions

In summary, we proposed an integrative analysis based on the gene expression profiles and the reconstructed protein-protein interaction network for prostate cancer, in contrast to the conventional methods of examining differential genes expression or proteins expression. In particular, this study was more intensive analysis on modular analysis for investigating the progression of different disease phases of prostate cancer. The achieved significant modules resulted in the identification of the candidate disease-associated genes which were consequently regarded as potential module biomarker. It can be effectively used as the promising feature to distinguish between control and disease samples. Regarding on functional analysis of candidate disease-associated genes, interestingly a major fraction of genes was enriched in the nucleus and different functions were encoded for transcription factors. Concerning on pathway enrichment analysis, Transcription Androgen Receptor (AR) nuclear signaling and Epidermal Growth Factor Receptor (EGFR) signalling pathway were clearly shown to be the pathway targets for prostate cancer progression. Transcription factor AR plays an important role in Transcription relationship and acts as a hub for regulating a lot of genes in the Transcription AR nuclear signaling. EGFR signalling regulates cell proliferation, cell differentiation, cell cycle, and cell migration and therefore it has been a potential interested target for effective cancer therapies. Last but not least, we successfully found an interesting transcription factor Sp1 which could be regarded as a potential novel biomarker for prostate cancer. For a future work, we will further study the experimental validation of potential disease genes and pathways during prostate cancer progression.

## Competing interests

The authors declare that they have no competing interests.

## Authors' contributions

YL performed integrative data analysis. WV analyzed the gene expression data. YL and WV wrote the paper. and wrote the paper. BS and LC conceived and designed the overall study and revised the manuscript.

## Supplementary Material

Additional file 1 **Supplementary file 1**. All the Supplementary Figures and Tables mentioned in the paper Click here for file

Additional file 2 **Supplementary file 2**. The reconstructed HPC-PPIN with annotated functions is shown in it. Click here for file

Additional file 3 **Supplementary file 3**. A graphic representation of HPC-PPIN by Cytoscape is presented in it. The file is in .cys format which can be opened by Cytoscape software. Click here for file

## Declarations

The publication costs for this article were funded by National Natural Science Foundation of China (NSFC) (grant no. 31170795 and no. 91230117).

This article has been published as part of *BMC Medical Genomics *Volume 7 Supplement 1, 2014: Selected articles from the 3rd Translational Bioinformatics Conference (TBC/ISCB-Asia 2013). The full contents of the supplement are available online at http://www.biomedcentral.com/bmcmedgenomics/supplements/7/S1.
