# Supplementary material for: Integrative analysis reveals disease-associated genes and biomarkers for prostate cancer progression
Source: BMC Med Genomics. 2014 May 8;7(Suppl 1):S3. doi: 10.1186/1755-8794-7-S1-S3 (PMC4110715; doi:10.1186/1755-8794-7-S1-S3)
Supplement: Additional file 1 — Supplementary file 1. All the Supplementary Figures and Tables mentioned in the paper [file 1755-8794-7-S1-S3-S1.doc]

# Supplementary file 1

**B**

**
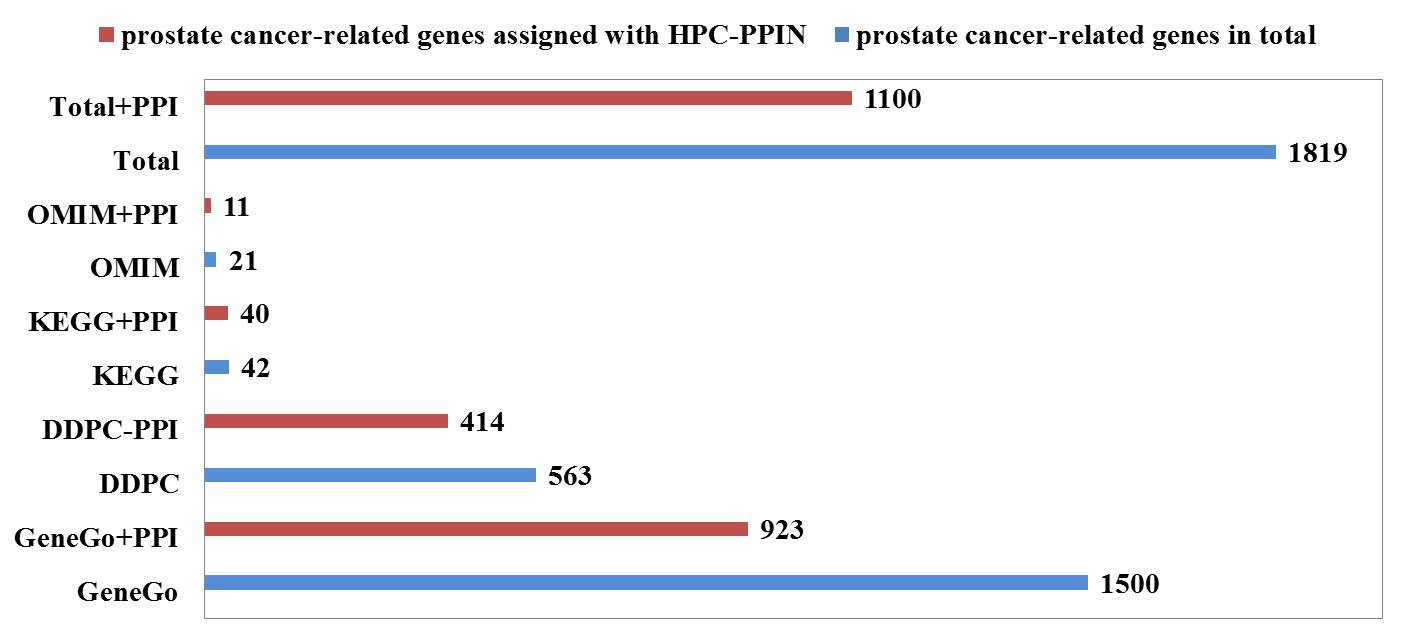
**

**Figure. S1** **(A)** Pie chart shows distribution of prostate cancer-related genes from different databases with assigned and unassigned in HPC-PPIN. (B) Bar chart shows prostate cancer-related genes assigned in HPC-PPIN and prostate cancer-related genes in total.

**
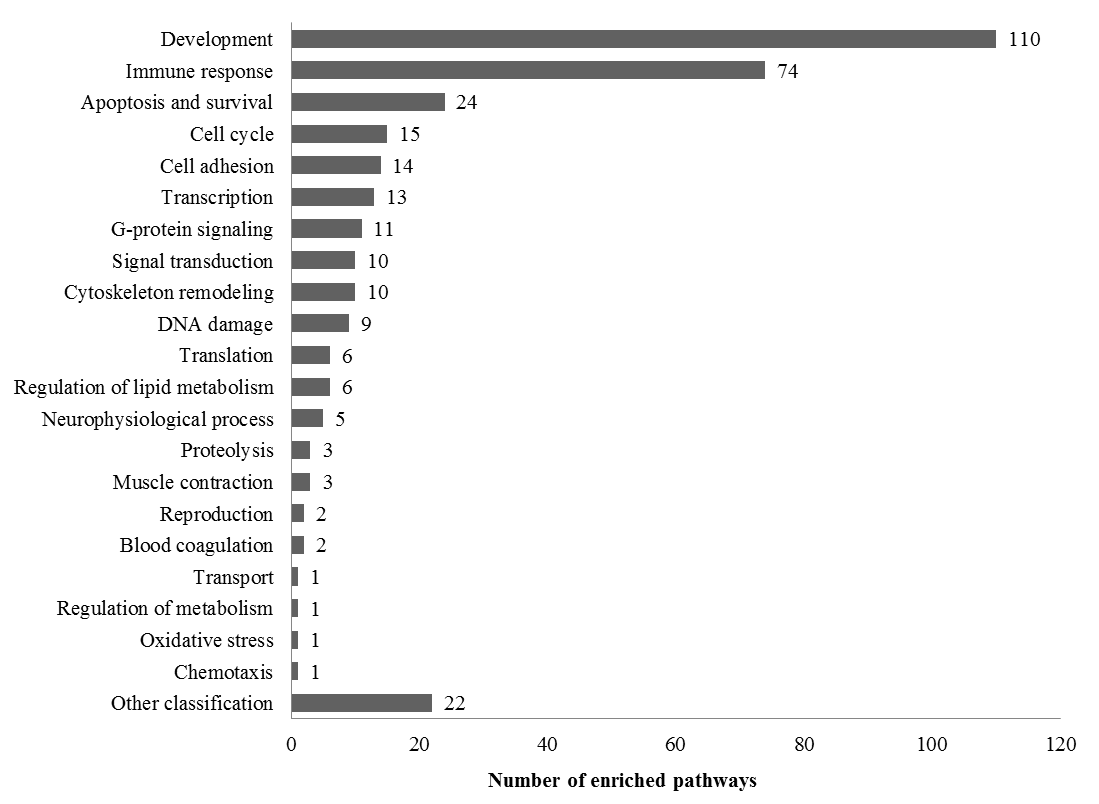
**

**Figure. S2**: GeneGo Ontology classification of 344 enriched pathways from candidate disease-associated genes shared by three disease phases. Bar chart shows these enriched pathways were distributed into 22 different categories.

**
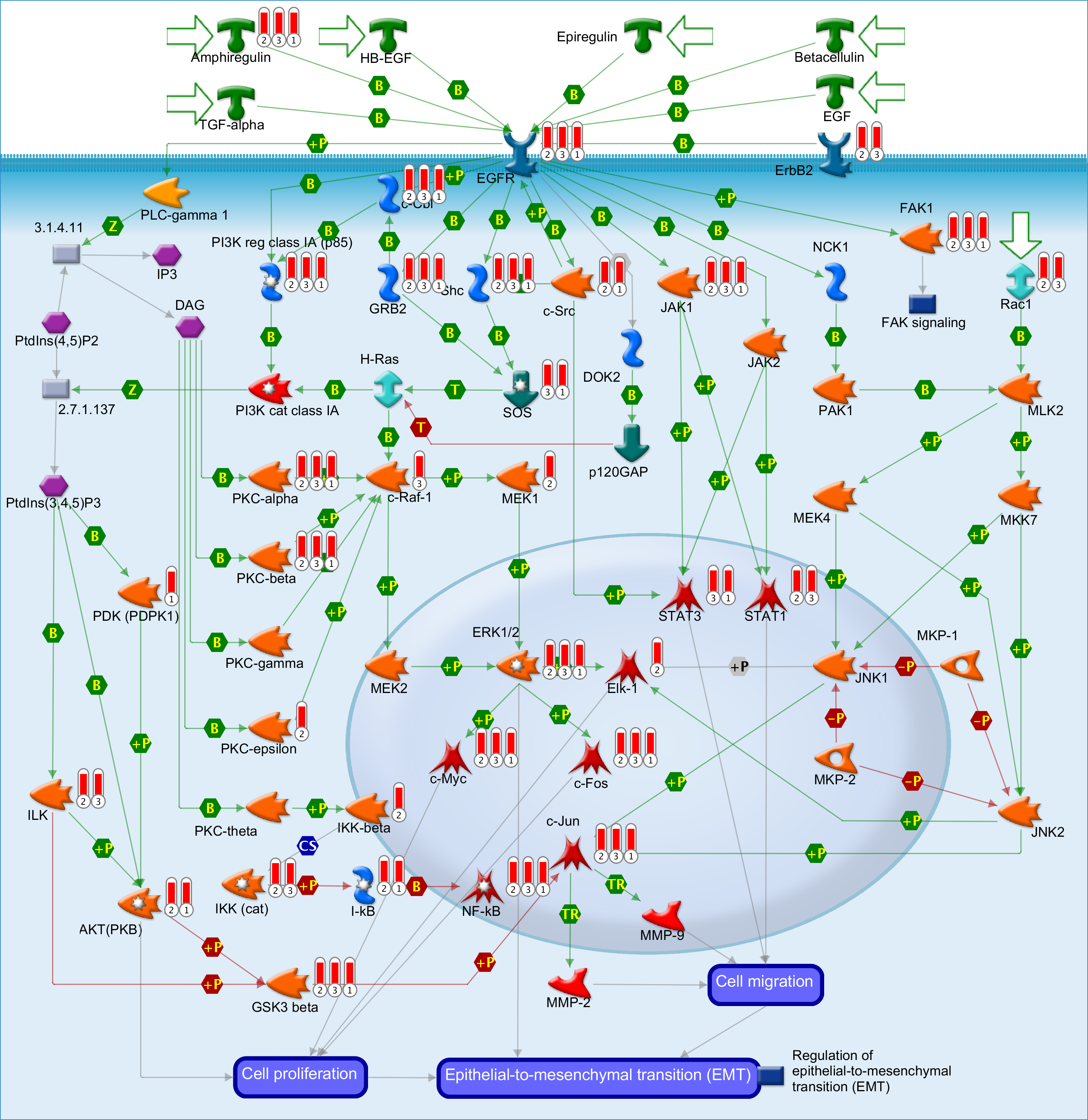
**

**Figure. S3** GeneGo graphic illustration represents [Development EGFR signaling pathway](http://portal.genego.com/cgi/exp_stat.cgi?diagram_type=maps&channel=Menu&section=Tools)

**(A)**

**
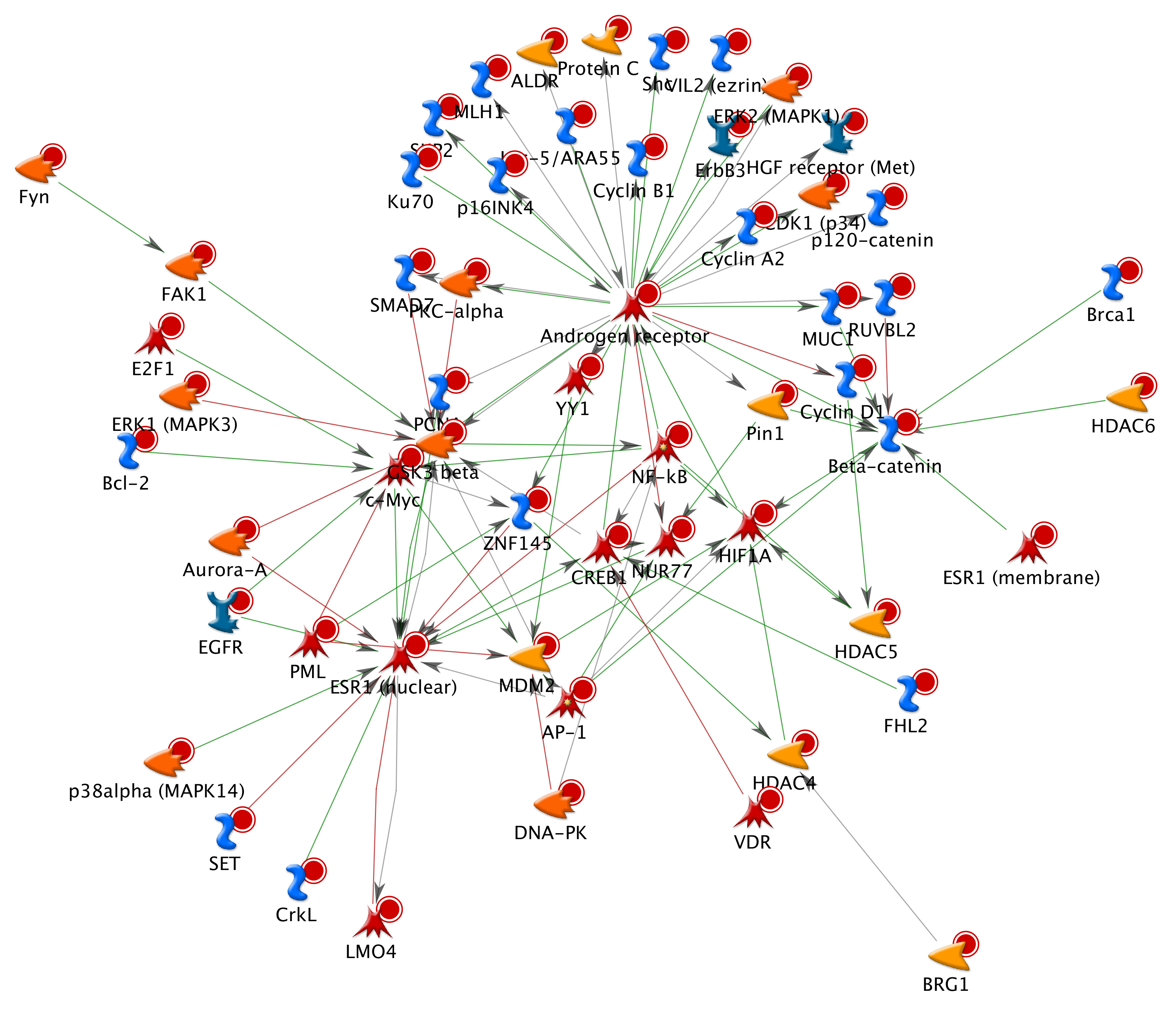
**

**(B)**

**
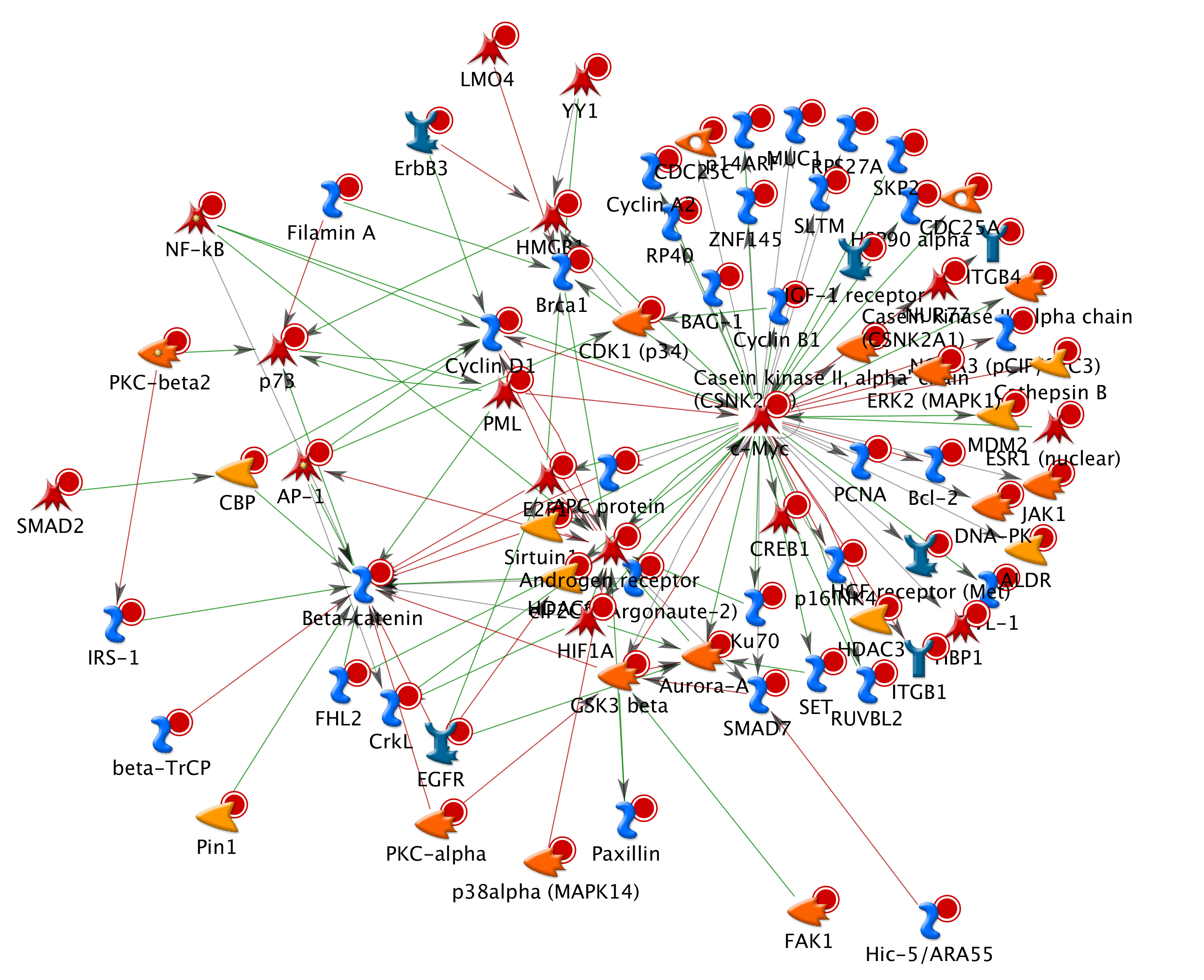
**

**(C)**

**
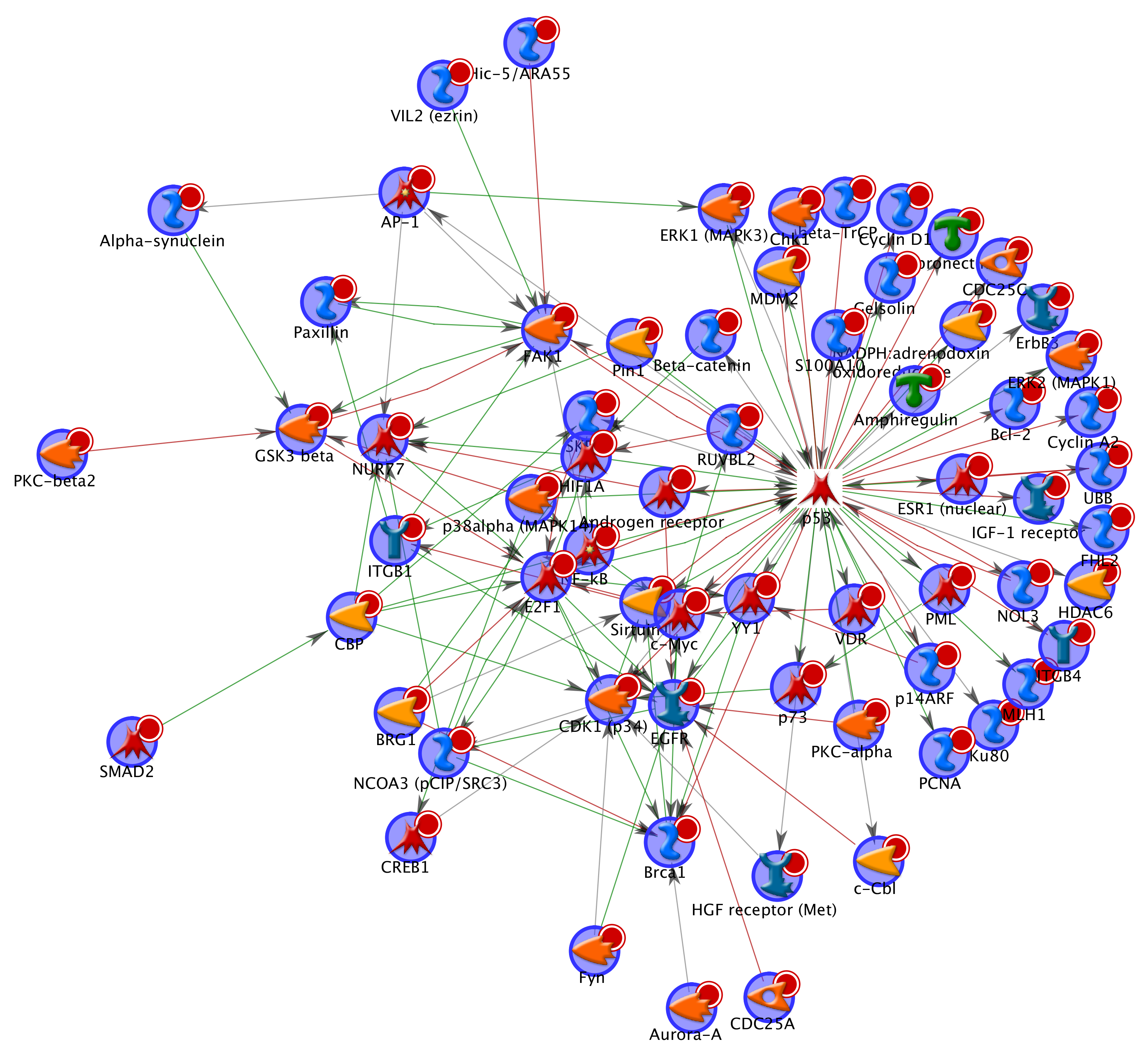
**

**Figure. S4** GeneGo graphic illustrations represent key transcription factors which regulate a lot of candidate disease-associated genes identified in the TF regulated network (A) Transcription factor AR, (B) Transcription factor MYC, (C) A hidden key transcription factor p53.

**Table S1: List of all unique gene expression signatures identified in each module**

| **Pairwise disease phases comparison** | **Number of unique gene expression signatures** | **List of unique gene expression signatures** |
| --- | --- | --- |
| Early-Middle phases | 56 | TXN,CD40,HSPA5,MAPK3,SYK,BMPR1B,CASP9,DCC,FBXW7,GAPDH,HCFC1,HDAC5,HSP90B1,HSPA9,KLF6,MSX2,NCOA2,PDGFRB,PDPK1,PPARGC1A,THRB,TXNIP,ADRBK1,ATRX,BCL2L1,BIRC5,CCNA1,CDKN2C,COL1A1,CTNNBIP1,DAPK1,DHFR,FDFT1,FHL1,FKBP4,GATA2,HSPD1,JUND,KLF5,LRP1,MKI67,MYD88,NFYC,NOTCH1,NPAS2,PECAM1,PKD1,PRKCD,SOS1,SQSTM1,SREBF2,TGFB3,THBS1,TUBB3,UBE3A,XBP1 |
| Middle-Late phases | 30 | ANXA4,APLP1,ATF3,CCL2,ESR2,FBLN1,IKBKB,JUNB,PDLIM5,PTGS2,ATM,BCAR1,CALD1,EIF3H,ELK1,FSCN1,GADD45A,IGFBP3,INSR,ITGB2,LEF1,MAP2K1,MSH2,PRKACA,PRKCE,PRKD1,RPL10,SERPINA1,TIMP3,TYK2 |
| Early-Late phases | 82 | CD44,PTEN,ANXA1,BMPR2,BRAF,DDX5,ERBB4,GJA1,HNRNPK,ITGA5,MME,NCOA4,PGR,RARB,SPP1,TP63,WHSC1,A2M,ACVR1,ANG,APOE,ARAF,ASAP1,BMP6,BMP7,CCL5,CCND2,CDC20,CDH11,CDH3,CYP3A4,DLG1,E2F3,E2F4,EDNRA,EPOR,ERCC5,FABP3,FANCD2,FASLG,HDAC7,HGF,IFNAR1,IGF1,IGFBP5,IGFBP7,ISG15,ITGA3,KRT14,LAMA3,LEP,LIFR,LPL,LRIG1,LRP2,LYZ,MAF,MIB1,PDCD4,PNP,PRDM2,PRKG1,PTPRC,RAD9A,RAF1,SDC2,SEPP1,SMARCE1,SRF,STAT5B,STMN1,TBX3,TCEB1,TGFB2,TMBIM6,TNFRSF10B,TNFSF10,TOP1,TP53BP1,TRIM25,VCP,XIAP |
